# Is Neurolaw Conceptually Confused?

**DOI:** 10.1007/s10892-014-9168-z

**Published:** 2014-05-07

**Authors:** Neil Levy

**Affiliations:** Florey Institute of Neuroscience and Mental Health, University of Melbourne, Parkville, 3010 Australia

**Keywords:** Addiction, Law, Lie detection, Neuroscience, Psychology

## Abstract

In *Minds*, *Brains*, *and Law,* Michael Pardo and Dennis Patterson argue that current attempts to use neuroscience to inform the theory and practice of law founder because they are built on confused conceptual foundations. Proponents of neurolaw attribute to the brain or to its parts psychological properties that belong only to people; this mistake vitiates many of the claims they make. Once neurolaw is placed on a sounder conceptual footing, Pardo and Patterson claim, we will see that its more dramatic claims are false or meaningless, though it might be able to provide inductive evidence for particular less dramatic claims (that a defendant may be lying, or lacks control over their behavior, for instance). In response, I argue that the central conceptual confusions identified by Pardo and Patterson are not confusions at all. Though some of the claims made by its proponents are hasty and sometimes they are confused, there are no conceptual barriers to attributing psychological properties to brain states. Neuroscience can play a role in producing evidence that is more reliable than subjective report or behavior; it therefore holds out the possibility of dramatically altering our self-conception as agents and thereby the law.

In *Minds*, *Brains*, *and Law*, Pardo and Patterson (2013) set out a case for rethinking the conceptual foundations of neurolaw. Neurolaw is a broad movement which claims that neuroscientific findings are relevant to how the law is or should be made and applied; proponents claim that it will enable us better to determine guilt, or to deter (potential) offenders, or even that it will revolutionize the foundations of the law by leading to a new understanding of morality or responsibility. Pardo and Patterson (2013: 21) are far from skeptical of the claim that neuroscience can inform the law, for instance by providing evidence that an agent lacked control over his or her actions, or—more speculatively—that there is a high probability that a witness is attempting to deceive the court. For interlinked *conceptual* reasons, however, they believe that neurolaw has not yet made a convincing case for particular applications of neuroscience in the courtroom, and they reject the more dramatic claims made on its behalf. Neurolaw as it is currently understood is vitiated by important conceptual confusions, they claim, which it inherits from neuroscience as it is currently practiced. These conceptual confusions ensure that many of its claims for the legal relevance of neuroscience are, as a matter of fact, false; conceptual clarity, moreover, would allow us to see that the more dramatic claims made on behalf of neurolaw are *necessarily* false. Neurolaw enthusiasts claim, for instance, that the brain stores knowledge and that therefore we can develop a neuroscientific method of detecting (say) guilty knowledge which bypasses the need for testimony. This claim is false, Pardo and Patterson argue: not just as a matter of fact, but because the brain is not the kind of thing that can store knowledge.

The project Pardo and Patterson undertake is therefore essentially negative, though it is envisaged as a necessary prolegomenon to a positive project: the sober and careful use of neuroscientific findings in the courtroom. The negative project consists in identifying the conceptual errors made by neuroscientists and enthusiasts for neurolaw. The positive project, to which they have space to do no more than gesture, is to rethink neurolaw once the conceptual errors have been identified and rejected. In this response, I will argue that though some of the conceptual confusions that Pardo and Patterson identify are genuine, these confusions are neither as numerous nor as significant as they suggest. The central confusion they allege—mistaking neural properties for psychological properties—is no confusion at all, I will suggest. The argument for this claim will constitute the bulk of the paper, and will focus (following Pardo and Patterson’s lead) on conceptual issues concerning whether neuroscience *can* inform the law, rather than on concrete suggestions as to just how it might. In the last substantive section, I will turn to this kind of question, to show how neuroscience could (justifiably) have a significant impact inside the courtroom. I will sketch a hypothesis concerning how addiction works to alter the beliefs of addicts; a hypothesis that, if true, would be of direct relevance to assessing the degree of culpability of addicts. While the hypothesis is speculative, it is meant only to be illustrative; we ought to expect neuroscientific findings like it to significantly alter our assessment of agents and their actions.

## Conceptual Errors in Neurolaw?

Pardo and Patterson see neurolaw as it is currently practiced as founded on a thoroughgoing reductionism: the reduction of mind to brain and of mental processes to neural processes. In the place of this reductionist program, they want to assert the primacy of proper criteria for the ascription of mental properties to agents. The mind, they assert, consists in a set of powers, capacities and abilities, and the correct criteria for ascription of these powers, capacities and abilities are behavioral. Behavior provides privileged criteria—what they also call the "measure"—for the ascription of mental properties. Neuroscience can at most provide a *measurement* for these properties, where a measurement provides evidence that a concept applies, rather than a definitive ruling on its applicability. When we recognize the primacy of these behavioral criteria, they claim, we come to see that many of the claims made on behalf of neurolaw are (literally) nonsensical, and its ambitions either as yet unsatisfied or, often enough, unsatisfiable. In response, I will argue that *neither* brain nor behavior provide privileged criteria for the ascription of mental states. There are not such criteria: rather any explanatory level may be revised in the light of any other. In some cases, we may end up asserting the primacy of behavior; in others, neural (or evolutionary, or cognitive psychological, perhaps even sociological) evidence will trump behavior. In most cases, no level will be privileged, and assigning content to states will be a matter of seeking the best fit between all explanatory levels at once.

The central conceptual error identified by Pardo and Patterson is what they (following Bennett and Hacker 2003) call the *mereological fallacy*. Someone commits the mereological fallacy when they attribute to brains or to parts of brains properties that belong to agents alone (for instance, when someone says that “the brain decides”). The other alleged conceptual errors they identify are typically particular instances of or at any rate closely related to the same fallacy: mistaking the brain for the mind or thinking that brains can engage in activities like rule-following. Philosophers of mind and neuroscientists often dismiss such talk as metaphorical or harmless analogizing. Pardo and Patterson (2013: 46–53) think that these conceptual errors are not harmless; rather, they cause trouble when neuroscience is applied to legal questions. For instance, they may lead to mistaken views about the nature of jurisprudence; more consequentially, they may lead to mistaken normative claims (Pardo and Patterson 2013: 53–63). I will suggest that though these fallacies may sometimes cause harm, such instances are rare (as indeed is the commission of the fallacy itself). Sometimes the talk is genuinely illuminating of the phenomena described. Sometimes no fallacy is committed because the claims Pardo and Patterson identify as conceptually confused are in fact literally true.

Let me begin with what Pardo and Patterson (2013: 7) take to be the central cause of the errors made by enthusiasts for neurolaw: reductionism. They define reductionism very broadly: someone counts as a reductionist if they subscribe either to an identity claim—the mind *is* the brain—or to the explanatory claim that mental attributes can be fully explained “in terms of information about the brain”.^1^ Since I have no wish to defend the mind/brain identity thesis, let me focus on the explanatory claim. It is the explanatory claim that must do the heavy lifting in neurolaw, after all. Whether the brain is identical to the mind or not is of no *direct* interest to the law; it is only if the identity claim entails (or supports) functional claims that it becomes relevant. Indeed, Pardo and Patterson think that some kinds of identity claims underwrite the explanatory claims: not the identity of mind (tout court) with brain, but of mental processes with brain processes.

Their opponents, they claim, might be seen as neuro-Cartesians: though neurolaw enthusiasts reject substance dualism (which Pardo and Patterson rightly reject as a red herring in this debate), they merely substitute the brain for the Cartesian *res cogitans*.^2^ For the (neuro-) Cartesian view they substitute an Aristotelian view, according to which the mind is not a substance; rather it is “an array of powers, abilities, and capacities” (Pardo and Patterson 2013: xxvi). This claim is absolutely central to Pardo and Patterson’s case against neurolaw as it is currently practiced, because they take it to entail the primacy of behavior when it comes to attributing mental states to agents. And that, in turn, they take to entail that only to the conscious person can mental states and processes appropriately be attributed. From this fact, they argue, it follows that a number of the claims made by neuro-reductionists are false (that we can see thoughts in the brain; that the brain engages in deception; that there can moral principles instantiated in the brain, and so on).

I am unsympathetic to Pardo and Patterson’s Aristotelian conception of the mind: In common with most other people in cognitive science, I identify the mind with the totality of information processing mechanisms that underlie the powers and capacities on which they fix.^3^ However, I will not attempt to argue directly for the superiority of my view over theirs. Rather, I aim to show that the Aristotelian conception that the mind consists in a set of capacities does not entail that the exercise of these capacities provide a privileged measure for particular mental states.

Pardo and Patterson argue that the Aristotelian conception of the mind entails that the exercise of the powers and capacities we associate with being minded (perception, sensation, memory, and so on) is the measure of correct attribution of these mental states and processes to people. The strong primacy of behavior does not entail that when there is no behavior there is no mentation—Pardo and Patterson are not behaviorists—but it does entail that neuroscience can produce only a measurement of mentation, not a measure of it. This enthrones behavior as the uniquely privileged (though defeasible) measure: When there is a clash between behavioral evidence and science, the science must always give way. In turn, this entails that no brain process can correctly be identified with anything stronger than a correlate and a cause of mentation, never with a mental process *itself*. More significantly, it entails that the conscious agent is the only appropriate subject of mental states, and that in turn entails that there cannot be unconscious rule-following or a moral grammar instantiated in neural states.

There are two interlinked ways to block the inference from the Aristotelian conception of mind to the strong primacy of behavior and the related claim that mental processes can only be correctly ascribed to the conscious subject. The first turns on the claim that it is generally a mistake to attempt to read off metaphysics from semantics. We might accept some version of the claim that behavior is the measure of the mind, but from the (supposed) fact that the exercise of the powers and capacities we associate with being minded constitute our criteria “for our attribution of mental attributes” (Pardo and Patterson 2013: 3) it does not follow that these mental attributes are *constituted* by the exercise of these powers and capacities. How we know something is one thing; what it actually is quite another. The fact that semantics is an unreliable guide to metaphysics entails that Pardo and Patterson’s preferred means of blocking identity claims, by reference to what it is appropriate to *say*, fails.

Consider, for instance, their claim that knowledge cannot be a brain state. In the context of neurolaw, this claim matters because if knowledge is a brain state we might be able to use (for instance) fMRI evidence to demonstrate a guilty mind in the courtroom, whereas if knowledge cannot be a brain state, the neuroimaging data might have evidential value but it would be less powerfully persuasive: The evidence would be highly defeasible, since it would be an indirect measurement of a mental state and not a direct measure of its presence. Pardon and Patterson deny that knowledge is a brain state by reference to the fact that in some contexts substituting “my brain is in state S” for “I know that X” seems to alter the meaning of sentences in which the second phrase occurs. Their example is this: whereas the sentence “I know that X and X is false” is apparently nonsensical, “my brain is in state S and X is false” is a perfectly sensible sentence. If the two sentences differ in meaning, then the phrases that constitute the difference between them must also differ in meaning. Hence “my brain is in state S” has a different meaning to “I know that X”, and therefore *knowing that X* cannot be identical to *brain state S*; since the argument goes through for any brain state, *knowing that X* cannot be identical to a brain state (Pardo and Patterson 2013: 139–140).^4^

This argument fails because it attempts to read off what must be the case from what it is appropriate to say, and thereby attempts to draw metaphysics from semantics. What it seems appropriate to say depends on how things seem to us, but the reference of words may depends on facts other than how things seem to us (we shall see how this can be true shortly). Consider a famous example of G. Frege’s, distinguishing connotation and denotation. Frege points out that no person who is minimally rational could deny that the following sentence is true:

1. Hesperus is Hesperus.

But it seems perfectly appropriate to deny that the following sentence is true:

(2) Hesperus is Phosphorus.

Indeed, prior to Pythagoras, all knowledgeable people would have denied that sentence (2) is true. In fact, we might imagine a contemporary of Pythagoras utilizing some version of Pardo and Patterson’s argument against him (though the argument would be more convincing if we substitute Frege’s original descriptive language for the proper names “Hesperus” and “Phosphorus”): While “the evening star is the evening star” is clearly true, “the evening star is the morning star” is nonsensical! Of course, Pythagoras’s contemporary would have been wrong: Hesperus *is* Phosphorus. The moral of the tale is we cannot read off identity claims from linguistic propriety, or metaphysics from semantics.

The second way to block the inference from the Aristotelian conception of the mind to the claim that behavior is the measure of the mind is via the metaphysics of dispositions. Powers, abilities and capacities are dispositions (Vihvelin 2004). Objects—and organisms—possess dispositions in virtue of having sets of properties upon which the dispositions supervene. For instance, ‘fragility’ is a disposition (roughly, the disposition to shatter when struck in appropriate conditions) which vases often possess, in virtue of certain facts about the bonds between the molecules that constitute them. Mental powers, too, are dispositions, and we humans possess these dispositions in virtue of sets of physical properties. Neuroscience can study these physical properties, and it can identify the disposition with these properties. At least, the mereological fallacy is no barrier to doing so (notice that there can be conceptual barriers to the identification of the mind with brain which do not preclude identifying particular powers with neural states and processes).

Let us see how we can block the move from the primacy of behavior to the identification of mental states with neural states. We begin by accepting, with Pardo and Patterson, that behavior gives us the criteria for the attribution of mental states. As they point out, a putative claim that lying was identical to a particular brain state would be falsified by a clear example of someone telling the truth while in that brain state. However, it does not follow from this that behavior provides a strongly privileged measure of lying or any other mental process. Rather, the behavior *fixes the reference* of the term. The idea here is originally owed to S. Kripke’s discussion of natural kinds (Kripke 1980). Whether or not mental processes are natural kinds—Pardo and Patterson deny that they are (Pardo and Patterson 2013: 115), though they do not offer an argument for the claim—it is useful to model our discussion on Kripke’s. Reference fixing by ostention or description—*that stuff over there*; that is what I mean by ‘gold’; *retrieving past experience*; that is what I mean by ‘memory’—does not establish the measure for the property we are talking about; it is only the first step in investigating it.

The second step may involve scientific experimentation. In the case of ‘gold’, it ultimately involved discovering its atomic number. In the case of ‘memory’ it involved careful experimentation which revealed that memory fractionates into a variety of subsystems—working memory, procedural memory, semantic memory—and the discovery that performance is subject to characteristic limits and degradation; more interestingly, it led to the discovery of conditions under which memory is more and less reliable and the dissociation of subjective certainty from objective performance. In the course of this investigation, and especially given the dissociation between subjective certainty and objective performance, we may reasonably come to regard certain neural processes as better criteria for the possession or the lack of memory than either behavior or testimony: substituting for Pardo and Patterson’s behavioral criteria neural criteria.^5^

Pardo and Patterson (2013: 102) argue against this possibility by the following thought experiment: Suppose a person evinces brain activity “that is purported to be knowledge of a particular fact about a crime” but cannot “engage in any behavior that would count as a manifestation of that knowledge”. On what basis “could one claim and prove that the defendant truly has knowledge of this fact?”. We have already seen the dangers of attempting to draw one’s metaphysics from semantics. Let us fight thought experiment with thought experiment to see how this argument fails. We will substitute for “memory” a standard Kripke-style example: water.

The description “the stuff in rivers and lakes that we drink, which freezes at 0 °C and boils at 100 °C” fixed the reference of our word “water” and its translations. But scientific investigation revealed that “water” was identical to a chemical compound consisting of hydrogen and oxygen in a particular ratio. Water = H_2_O. The reference-fixing description is no longer the measure of water; rather, the scientific formula has at least as good a claim on being the measure. Suppose we found some H_2_O that did not freeze at 0 °C (in otherwise appropriate conditions). Should we think that it is not “water” at all? Some philosophers think the answer is “no”; the scientific claim provides the measure. I think an equally defensible answer is “not necessarily”; *neither* the functional definition *nor* the scientific provides a definitive measure. Rather, we may have a decision to make; one guided by our interests and concerns and the further details of the case. Sometimes the scientific criteria will have a strong claim to be the measure; sometimes some other criteria will be a better measure, and sometimes no set of criteria will constitute a measure.

Exactly parallel things can be said about “memory” as about “water”. There is no reason to think that the functional description provides a strongly privileged measure of memory and therefore no reason to privilege behavior. A case might be made for scientific criteria superseding the functional; depending on the details of the science, though, it may be that a stronger case can be made for neither functional nor scientific description being a privileged measure. In either case, we may quite appropriately be able to say of the person who sincerely denies any knowledge of a past event that nevertheless she possesses the memory (why not, after all? Possession is one thing, access another, as anyone who has experienced the tip of the tongue phenomenon should know).

Pardo and Patterson (2013: 112) accept that neural criteria *can* be substituted for behavioral criteria. But, they claim, that would involve changing the meaning of the concepts. The law, they say, is usually interested in “the behavioral and psychological phenomena currently referred to by the terms expressing these concepts”, not in some putative successor concepts. Moreover, these phenomena will persist, even if the reference of our terms “knowledge”, “intention”, “lies” and so on alters, and in most cases it will continue to be these concepts in which the law will be interested. The law cares about *lying*, *intentions*, and so on, not in putative successor concepts picked out by neural criteria.

Conceptual change like that envisaged by Pardo and Patterson can indeed occur, though in cases like this the older concept is typically eliminated: the new concept replaces the old because the new concept is seen to be preferable (more useful, or to do a better job of picking out something in the world) to the old. Elsewhere, I have made a case for the elimination of the psychological concept “weakness of will” (Levy 2011). But conceptual change need not be the upshot of scientific discoveries. If—to use their own example—“depression” comes to be identified with a chemical state of the brain, this will probably be because we have discovered something about the *referent of our term*, not because we have eliminated “depression” from our psychological vocabulary. In actual cases in which concepts have been eliminated by advances in knowledge, moreover, the older phenomenon referred to has not persisted alongside the new concept: Rather, the advance has consisted in demonstrating that *it never existed at all* (think of “witches” or “phlogiston”). If weakness of the will is eliminated, as I propose, it will be because no one has ever actually been weak willed: Rather, the behavior in which they have engaged and that we have hitherto described as “weak willed” will be shown to be better described utilizing some other vocabulary. In cases in which neuroscience reveals that some psychological state has properties of which we are currently ignorant, or eliminates some psychological property altogether, the law will continue to use the old concepts at the cost of refusing to recognize the actual psychological properties of real people.

The behavioral criteria that Pardo and Patterson emphasize indeed play a special role: that of reference fixing.^6^ Once we have the reference of our terms fixed, we can begin to investigate them further. We may discover that the terms refer to states or processes that have essences. We may discover that that they refer to a motley of states, some of which are better candidates for being the psychological state with which we started than others in which case the neural evidence might lead to the refinement of our concept. Or, we may find that nothing answers well to our concept, in which case we will look for a successor concept, or eliminate it. With sufficient convergent evidence—from neuroscience, cognitive and social psychology, and even from evolutionary theory and ethology—we may have excellent reason to think that our original reference fixing behavioral criteria do a much worse job in tracking the genuine psychological states of agents than some set of scientific criteria (Shea and Bayne 2010).

## The Wittgensteinian Framework in the Light of Cognitive Science

The framework for understanding the mind that Pardo and Patterson adopt is explicitly and self-consciously Wittgensteinian. But the Wittgensteinian framework is inadequate for cognitive science. It has, quite simply, been rendered antiquated by developments over the past five decades. L. Wittgenstein was concerned to avoid what in retrospect can be seen to be the homunculus fallacy: the fallacy of offering an explanation of a psychological capacity by postulating an entity that possesses the very capacity that needs explaining. If, for instance, we ‘explain’ visual perception by postulating a mechanism inside the brain (or the mind) that views images projected through the retina, we seem to commit the fallacy: We have ascribed to the mechanism the very powers (of visual perception) we aimed to explain. Pardo and Patterson seem to think that we commit a subtler version of the same fallacy if we postulate that knowledge—in their primary example, memory—is stored in the brain. If there was such knowledge in the brain, they maintain, “people would *have to remember how to access the recording and interpret its content*”. Moreover, since people cannot see their own brains, this knowledge would be inaccessible to them (Pardo and Patterson 2013: 104). The basic point is clear: in postulating that the brain stores memories, we commit a version of the homunculus fallacy because we explain memory in terms that presuppose it. All the work of accessing memories remains to be explained, including how the person *remembers* how to access them.

Indeed there is a problem here. But it is a problem that neuroscience has solved. The real lesson of the homunculus fallacy is that when we explain things, we do so not by postulating mechanisms that have the very powers we want to explain, but postulating simpler mechanisms (indeed, this is very generally true: we explain the complex in terms of the simple). Memories can indeed be stored in the brain (and in fact we have a reasonably detailed understanding of just *how* memories are stored in the brain) without people needing to remember how to access them or needing to be able to see their brains. These high level capacities are explained by the workings of simpler mechanisms. The very base level mechanisms do not know, perceive, think or recall anything: they simply fire or fail to fire, in response to stimuli of the appropriate sort. Together some set of these mechanisms—neurons—constitute circuits that might be said to have glimmerings of perception or thought, or some other psychological property; in any case, they process states that can be said to have contents. Sets of these circuits constitute modules to which we can quite confidently attribute some psychological properties, though *some* of these properties fall far short of the high-level psychological properties, *some* of which are only appropriately ascribed to the person.

Consider the psychological property of deciding, which Pardo and Patterson (2013: 75–76) explicitly deny can rightly be predicated of the brain or of a part thereof. A decision resolves uncertainty about what to do. Uncertainty, in turn, can be understood as a state of being poised between alternatives, both or all of which are open prior to the decision (in ordinary language “uncertainty” often connotes a feeling of doubtfulness, but it is clear that no such feeling is an invariable accompaniment of decision-making, and therefore this is no barrier to attributing uncertainty to brain mechanisms). There is no reason why a modular mechanism cannot be uncertain in the sense defined. And there is no reason why we should not call the resolution of this uncertainty a decision. Doing so accords very well with the everyday meaning of “decision.” Why should we withhold the description? Not because of any fallacy; none has been committed.

We can, if we like, decide to restrict the use of the term “decision” to the personal-level. But there is no principled reason why we should use language this way, and there are very good reasons why we should not: *we illuminate both the personal level phenomenon* *and the subpersonal phenomenon* when we use the same term to refer to both. We illuminate the personal level phenomenon because, as a matter of fact, the decisions of persons are normally caused and perhaps even typically constituted by these subpersonal processes; we illuminate the subpersonal phenomenon because we draw attention to the fact that what is occurring at this level is recognizably an instance of a process of the same general kind as personal-level decidings.

In many other cases, the subpersonal processes are recognizably psychological but sufficiently different from personal-level psychological processes to warrant us using a different vocabulary. Consider the representational contents of states produced by lower levels of the visual system. They are genuinely representational: They have truth conditions and therefore can misrepresent. But they do not have many of the features that we associate with belief, especially sensitivity to domain-general information (of course, we can say the same of the representational properties of many modular systems). For that reason, we ought to hesitate before we call them ‘beliefs’. Calling these states beliefs threatens to mislead: not because we commit the mereological fallacy—attributing to the subpersonal a property that belongs only to persons—but because they lack many of the properties we associate with personal-level beliefs. Better to say that they are representational states and define how they differ from those higher-level representational states we call ‘beliefs’. No fallacy blocks our attribution; rather, a concern to get matters straight is what motivates us here, and that concern will only prevent us from using the psychological vocabulary in some cases and not others.

## Neurolaw in the Courtroom

Thus far the discussion has been very much rooted in the theoretical domain. It is now time to see what implications it has for the law. Pardo and Patterson believe that the conceptual confusions of neurolaw disqualifies it from playing a role in the courtroom today: Because it mistakes neural states for mental states, it advances its evidential claims with far more confidence than is warranted, and thus risks misleading judges and juries. For the most part, I agree that neurolaw is not ready for prime time: Apart from a relatively few cases, such as those in which a lesion significantly raises the probability of a defendant suffering from a disorder of control (cases in which Pardo and Patterson agree that neuroscientic evidence may be relevant), the research findings are not yet reliable enough to be utilized in high-stakes contexts. The reasons for this are manifold, and many of them are identified by Pardon and Patterson in their discussion of neuroscience-based lie detection: unrepresentative samples; variability across subjects; our ignorance concerning whether and how results may be manipulated by subjects; inadequate paradigms that diverge too significantly from what courts are interested in when they probe honesty, and so on.

While some of the problems identified—e.g. the mismatch between the paradigms utilized in the experiments and what courts are interested in when they ask about the honesty of witnesses—are indeed conceptual, none of these problems concern the basic assumptions made in neuroscience and neurolaw and none are insuperable barriers to the use of neuroscience in the courtroom. With further research, utilizing different paradigms and testing a wider range of subjects under a broader range of conditions, we can reasonably expect brain-based lie detection (to use Pardo and Patterson’s central example) to improve sufficiently to become admissible evidence in the courtroom.^7^ In this final section, I want to be more ambitious. I will sketch a (admittedly speculative) hypothesis, driven by contemporary neuroscience, which if true might transform how the law responds to crimes committed by addicts in pursuit of drugs.

For reasons of space, I will be brief. The kind of addict with whom I am concerned sincerely wishes to refrain from drug consumption (Pardo and Patterson might be reassured to know that our evidence for sincerity in these kinds of cases is often behavioral, consisting in the time and energy that addicts expend on avoiding drugs; see Ross et al. 2008). Why do people who judge that they ought to refrain from taking drugs nevertheless often find themselves using? Saying “because they are addicts” does not answer the question; it merely puts a label on it. The most common response to the question cites an irresistible urge. But there is plentiful—behavioral!—evidence that addiction does not cause irresistible urges (Levy 2012). Instead, I suggest that it causes *judgment shift*. Though most of the time addicts judge that they ought to refrain, at the time of consumption they judge that all things considered they ought to consume.

Note that this claim conflicts with the confident reports of at least some addicts who claim that at the very moment of using a drug they wholeheartedly judge that they should not use it. We should take first-person reports seriously, but they are just one piece of evidence among others: There is plentiful evidence that people can be wrong about their own mental states (consider the literature on affect misattribution, for instance, or on cognitive dissonance). We can have good evidence—which will almost certainly need to be convergent evidence, utilizing different methodologies and different kinds of measurements—that subjective reports are wrong. I suggest that this is one such case.

Addiction centrally involves a dysfunction of the mesolimbic system, which appears to be a value prediction system. When the system is working normally, it responds to cues predicting a reward, but not to the reward itself, once the relationship between cue and reward has been learned (Schultz et al. 1997). Because drugs of addiction directly drive up the dopamine signal, or otherwise cause the system to misfire, addicts cannot learn the relationship between drugs and reward. In effect, their dopamine system predicts that the drug will be more rewarding than ever, every time they take it (Hyman, 2005). This much is widely accepted. However, it remains controversial what role this dysfunction actually plays in the behavior of addicts.

There is independent evidence that the dopamine signal is a response to a violation of expectations, not merely to a predictor of (unexpected) reward (Corlett et al. 2004). It has the role of signaling a mismatch between the organism’s model of the world and the way the world actually is. It causes the organism to engage in some ‘behavior’ that narrows the gap between its prior model of the world and the actual world: this might be an action or it might involve changing the model of the world (or both). Now changing one’s model of the world is altering a doxastic state (I refrain from using the personal-level descriptor ‘belief’ not for fear of committing a fallacy but because we do not yet know enough about the content of the relevant states to know whether it is appropriate to call them beliefs). The dopamine signal therefore plays a role in changing the model of the world, and under the right conditions the update could filter up through the prediction error system causing a change in a (full-blown) belief. Because cues predicting the availability of a drug cause a pathological spike in dopamine firing, the addict may form the judgment that all things considered consuming is better than refraining (only to revert to her former judgment, that it is better to abstain, once she is removed from proximity to the cue).

The mechanism for judgment update sketched above is highly speculative. There is, however, evidence in its favor. There is evidence that mesolimbic dopamine plays a role in delusional belief formation (Corlett et al. 2006, 2007). Indeed, the model of belief update in addiction suggested here is modeled on Hohwy’s (2010) suggested mechanism for the processes underlying delusional beliefs. Because it remains speculative, however, I do not suggest that it ought to be cited in the courtroom. There is no *conceptual* barrier to its use: The problem rather is that right now we are not in a position to assert confidently that it is true. We need much more evidence, convergent evidence from different sources, both to ascertain the precise role of dopamine in belief formation and to rule out alternative hypotheses. The day may come, though, when it is appropriate to cite the hypothesis in the courtroom. We may be able confidently to assert that when she uses, or even when she engaged in a crime aimed at procuring money to buy a drug, there is a high probability that the addict acted as she judged she ought (all things considered).

If the hypothesis is correct, it would seem to make a difference to how courts ought to deal with addicts who have committed some (though by no means all) crimes that are directly or indirectly linked to their addiction. Yaffe (2013), who has on somewhat different grounds defended the claim that addicts frequently value what they are doing more than alternatives open to them at the time they commit some of the crimes linked to their addiction, has argued that there is a legal difference between behavior that is in accord with an agent’s values and behavior that bypasses her values. In the case of addicts who value how they act at the moment of acting criminally, compliance with the law would have required bearing a burden that reduces their responsibility, he suggests. Compliance requires them to bear the burden of acting contrary to their own values, at the moment of action, and this is a very substantial burden (though Yaffe remains uncommitted as to how much it reduces criminal responsibility).^8^

Obviously, since the science remains unsettled no court should accept either my account or Yaffe’s in mitigation right now. It is also obvious that much more work remains to be done before we can be confident in asserting that *even if one or other account is true*, it ought to reduce criminal responsibility. That work must be undertaken by legal scholars, in conjunction with scientists and philosophers. I (unlike Yaffe) lack the expertise to make a significant contribution to the legal debates. My aim has not been to establish that addicts have a partial defense; rather it has been merely to show that given current understandings of the science one may be available: one that it takes work in neuroscience (among other disciplines, ranging from philosophy to law) to reveal. The behavior of the addict is entirely equivocal as to whether she endorses her action at the moment she performs it; it is neuroscience that may allow us to settle the matter. Neuroscience, therefore, holds out the possibility of a dramatic transformation of the law in this case and perhaps in many others.

## Conclusion

There are no insurmountable conceptual barriers to neurolaw. Whether or not the mind should be identified with the brain (some) token mental states may be identifiable with neural states: Certainly, there are often good grounds to attribute representational content to neural states. Though I agree with Pardo and Patterson that right now there are relatively few neuroscientific findings that are sufficiently reliable for the courtroom, and none that should be transformative of the law, there are good grounds to expect that this will change. The dizzingly rapid progress of cognitive neuroscience, which provides evidence concerning how the brain processes information, over the past decade, and the enormous progress philosophers and psychologists have made in understanding how the low-level processes studied by neuroscientists map onto—or sometimes more interestingly, fail to map onto—the kinds of concepts of interest to the law (like belief, desire, intention, and so forth), provide strong grounds for believing that neuroscience will revolutionize our understanding of our minds, and thereby of ourselves as moral and legal agents.^9^

## Footnotes

1 At this point, many philosophers seem to have a legitimate complaint against Pardo and Patterson: they define “reductionism” so that some non-reductive physicalists count as reductionists. Now to their credit their definition is clear enough, so one may respond that they are entitled to use words as they like. However, it is dubious that they are entitled to this response: after all, one principal charge they lay at the door of those they allege to be conceptually confused is inappropriate use of language. Many of those they allege guilty of confusion explicitly signal that they intend to use the kind of language Pardo and Patterson warn against metaphorically. Pardon and Patterson evidently—and reasonably—believe that loose language can cause problems, because we may easily slip from using language metaphorically or stipulatively into taking it at face value. The same stricture should apply to them, though, as to their opponents. I note that they explicitly exclude me from the scope of what they call neuro-reductionism (Pardo and Patterson 2013: 2, n. 2), and they are right to do so: I neither identify the mind with the brain nor do I believe that we can fully explain cognition in terms of the brain. Nevertheless, my own views are clearly of the same family as those of the neuro-reductionists: like the reductionists, I claim that cognition can fully be explained in “causal, mechanical and non-volitional terms” (Pardo and Patterson 2013: 8); the difference between me and the neuro-reductionists consists principally in the fact that I claim the mind supervenes on (physical) properties external to the brain, as well as on the brain (see Levy 2007).

2 The claim that substance dualism is a red herring is indeed correct, and Pardo and Patterson rightly criticize me for introducing it. I think, however, we ought to go further and say that the accusation of Cartesianism itself as irrelevant. First, we might easily trade accusations here: it is far from obvious to me that the conception of mind they advocate is less Cartesian than that of mainstream neuroscience (after all, they retain the Cartesian insistence that mind has no location). Second and more importantly, the correct response to the accusation of Cartesianism—on both sides—is ‘so what?’ Cartesianism is widely rejected today because of specific problems, especially the interaction problem: how can an immaterial mind interact with a physical body? Neither side suffers from that problem, so it simply does not matter how Cartesian they are.

3 The information processing conception of the mind is preferable to the Aristotelian for precisely the kinds of reasons which led to the triumph of the cognitive revolution against behaviorism: because we understand mental processes only by attributing internal structure to the mind. Further, the computational metaphor, which sees cognition as processing over representations, has proved too fruitful to be lightly rejected.

4 As Robins and Craver (2011) point out, the use of “knowledge” in this context is misleading: knowledge cannot be a brain state because knowledge is a relation between a subject and a state of the world. Nevertheless, I retain Pardo and Patterson’s use of language for ease of discussion; in context it is apparent that no harm is done by the use of the word.

5 Substituting neural criteria for behavioral criteria does not entail substituting a non-Aristotelian conception of mind for Pardo and Patterson’s preferred view. Being minded might consist in having a set of capacities and powers, but the criteria for detecting mindedness might be neural, rather than behavioral. I thank a referee for pressing me on this point.

6 Patterson and Pardo argue that eliminativism can “never get off the ground” because research into the mind presupposes the very mental states it seeks to eliminate (Pardo and Patterson 2013: 107). But it presupposes these states only to fix the reference for further investigation; it is an open question whether further investigation will vindicate, refine, revise or eliminate the reference fixing criteria.

7 It should be noted, moreover, that *even if every one of Pardo and Patterson’s major claims were true*, brain-based lie detection might still be reliable enough for courtroom use. Even if it were a conceptual error to identify neural states with psychological states, even if we committed the mereological fallacy if we said that brains lie, even if brains could not follow rules, and so on, these facts would not prevent neuroscientific evidence being reliable. All that has to be true for its reliability is that neural states play an essential role in realizing psychological states, and that claim is completely uncontroversial (Robins and Craver 2011).

8 I identify an agent’s values with a set of relatively stable states to which she is committed, and therefore deny that addicts experience the kind of oscillation in values that Yaffe makes central to his claims. On my view, the burden borne by addicts is the burden of acting against their judgments, not their values. That might be a smaller burden, and therefore might be taken to mitigate legal responsibility to a smaller degree than the burden of acting against one’s values; on the other hand, it is an open question whether agents can rationally and intentionally act contrary to their own judgments.

9 One—especially important—example, among many. It has long been known that the folk psychological concept ‘memory’ fractionates into many different kinds, some of which are inaccessible to introspection (procedural memory, semantic memory, working memory, and so on; moreover, these of these systems themselves fractionate further). There is now increasing evidence that the folk psychological concept ‘belief’ fractionates in a parallel manner. We have attitudes that are encoded as associations between cues and natural rewards, systems that respond equally strongly to fictive feedback as to real, and systems that match outcome/action pairs to evaluate possible actions. These systems interact to drive behavior, but they sometimes conflict; in situations like this there may be a case for attributing conflicting beliefs to the person, even though neither their behavior nor their verbal assertions would support the claim. See Huebner (forthcoming) for documentation of the neuroscientific evidence for belief fractionation, as well as for reflections on how this fractionation matters for ethics.
